# A dock derived compound against laminin receptor (37 LR) exhibits anti-cancer properties in a prostate cancer cell line model

**DOI:** 10.18632/oncotarget.23236

**Published:** 2017-12-13

**Authors:** Charles Samuel Umbaugh, Adriana Diaz-Quiñones, Manoel Figueiredo Neto, Joseph J. Shearer, Marxa L. Figueiredo

**Affiliations:** ^1^ Department of Basic Medical Sciences, Purdue University College of Veterinary Medicine, West Lafayette, IN 47907, USA

**Keywords:** laminin receptor, drug discovery, prostate cancer, anti-cancer compound

## Abstract

Laminin receptor (67 LR) is a 67 kDa protein derived from a 37 kDa precursor (37 LR). 37/67 LR is a strong clinical correlate for progression, aggression, and chemotherapeutic relapse of several cancers including breast, prostate, and colon. The ability of 37/67 LR to promote cancer cell aggressiveness is further increased by its ability to transduce physiochemical and mechanosensing signals in endothelial cells and modulate angiogenesis. Recently, it was demonstrated that 37/67 LR modulates the anti-angiogenic potential of the secreted glycoprotein pigment epithelium-derived factor (PEDF). Restoration of PEDF balance is a desirable therapeutic outcome, and we sought to identify a small molecule that could recapitulate known signaling properties of PEDF but without the additional complications of peptide formulation or gene delivery safety validation. We used an *in silico* drug discovery approach to target the interaction interface between PEDF and 37 LR. Following cell based counter screening and binding validation, we characterized a hit compound’s anti-viability, activation of PEDF signaling-related genes, anti-wound healing, and anti-cancer signaling properties. This hit compound has potential for future development as a lead compound for treating tumor growth and inhibiting angiogenesis.

## INTRODUCTION

Laminin receptor (67 LR) is a 67 kDa protein derived from a 37 kDa precursor protein (37 LR) [1–3]. The derivation process most likely involves post-translational modification of 37 LR by acylation [3] or other lysine-directed conjugations [4, 5]. Because the two species are interrelated, laminin receptor at large is referred to as 37/67 LR. 37/67 LR is a member of the non-integrin receptor family [2]. 37 LR is encoded by the gene *RPSA* [6, 7], facilitates ribosome assembly [8], and is distributed to nuclear and cytoplasmic compartments of the cell [9]. Using laminin sepharose columns and/or tumor cells, several groups isolated and identified 67 LR [10–12]. 37/67 LR, perhaps owing to its laminin binding capacity, is frequently found at the plasma membrane [13–16]. Both 37 LR and 67 LR are able to bind laminin and laminin derived peptides [17, 18], molecules intimately involved in normal cell to matrix contact [19] and pathologically associated with cancer progression, metastasis, and invasion [20].

37/67 LR is regarded as a strong clinical correlate for progression, aggression, and chemotherapeutic relapse of several cancers including breast, prostate, and colon [21, 22]. The C-terminus of 37/67 LR has been suggested to increase viability and enhance survival of breast cancer cells [23]. In a lung cancer model, endogenous 37 LR expression levels were correlated with metastatic status, and overexpression of 37 LR enabled lung cancer cells to establish lung metastases *in vivo* [24]. Upregulation of 67 LR also has been associated with melanocyte tumor development [25], and invasiveness of ovarian and endometrial cancer [26, 27]. Downregulation of 37/67 LR by RNA interference (RNAi) inhibits cell migration and invasiveness [28], reduces cell binding to laminin and enhances the apoptotic response of colon cancer cells to doxorubicin [29], and inhibits hepatoma cell viability [30].

The ability of 37/67 LR to promote cancer cell aggressiveness is further increased by the ability of 37/67 LR to transduce physiochemical and mechanosensing signals in endothelial cells [31] and modulate angiogenesis [32] — a key component of the tumor/microenvironment interaction [33]. Recently, it was demonstrated that 37/67 LR modulates the anti-angiogenic potential of the secreted glycoprotein pigment epithelium-derived factor (PEDF) [34]. Originally discovered as a regulator of retinal and ocular health [35–37], PEDF is a non-inhibitory member of the serine protease inhibitor family that has been regarded as a novel and potent inhibitor of angiogenesis [38]. Administration or expression of full length PEDF or peptides derived from PEDF [39] markedly reduces vascularization [40] and endothelial cell viability [41] concomitant with a decrease in endogenous vascular endothelial growth factor (VEGF) [42].

Outside of angiogenic models, imbalance of endogenous PEDF has been observed in many cancers [43–45]. Restoration of PEDF balance is a desirable therapeutic outcome and has been achieved largely via gene delivery regimens [46–50] or through administration of PEDF peptides [39]. Additionally, 37/67 LR has been identified as an emerging and promising target for pharmacological intervention in cancer progression [51]. Therapeutic targeting to 37/67 LR has focused on the interaction between 37/67 LR and the green tea natural product, epigallocatechin gallate (EGCG) [52–54]. Acting through 37/67 LR, EGCG elicits anti-proliferative and anti-cancer effects in several cell lines [55–59]. Of high relevance to our study, a recent virtual based screen of National Cancer Institute (NCI) compounds against the laminin binding domain of 37/67 LR isolated several active chemicals with the ability to inhibit cell migration and binding to laminin at low micromolar doses [60].

Here, we used an *in silico* drug discovery approach to target the interaction interface between PEDF and 37 LR. Following cell based counter screening and binding validation, we characterized a hit compound’s anti-viability, activation of PEDF signaling-related genes, anti-wound healing, and anti-cancer signaling properties. This hit compound has potential for future development as a lead compound for treating tumor growth and inhibiting angiogenesis.

## RESULTS

### *In silico* screens produced a hit compound with promising antitumor activity in *in vitro assays*

**Figure 1:**
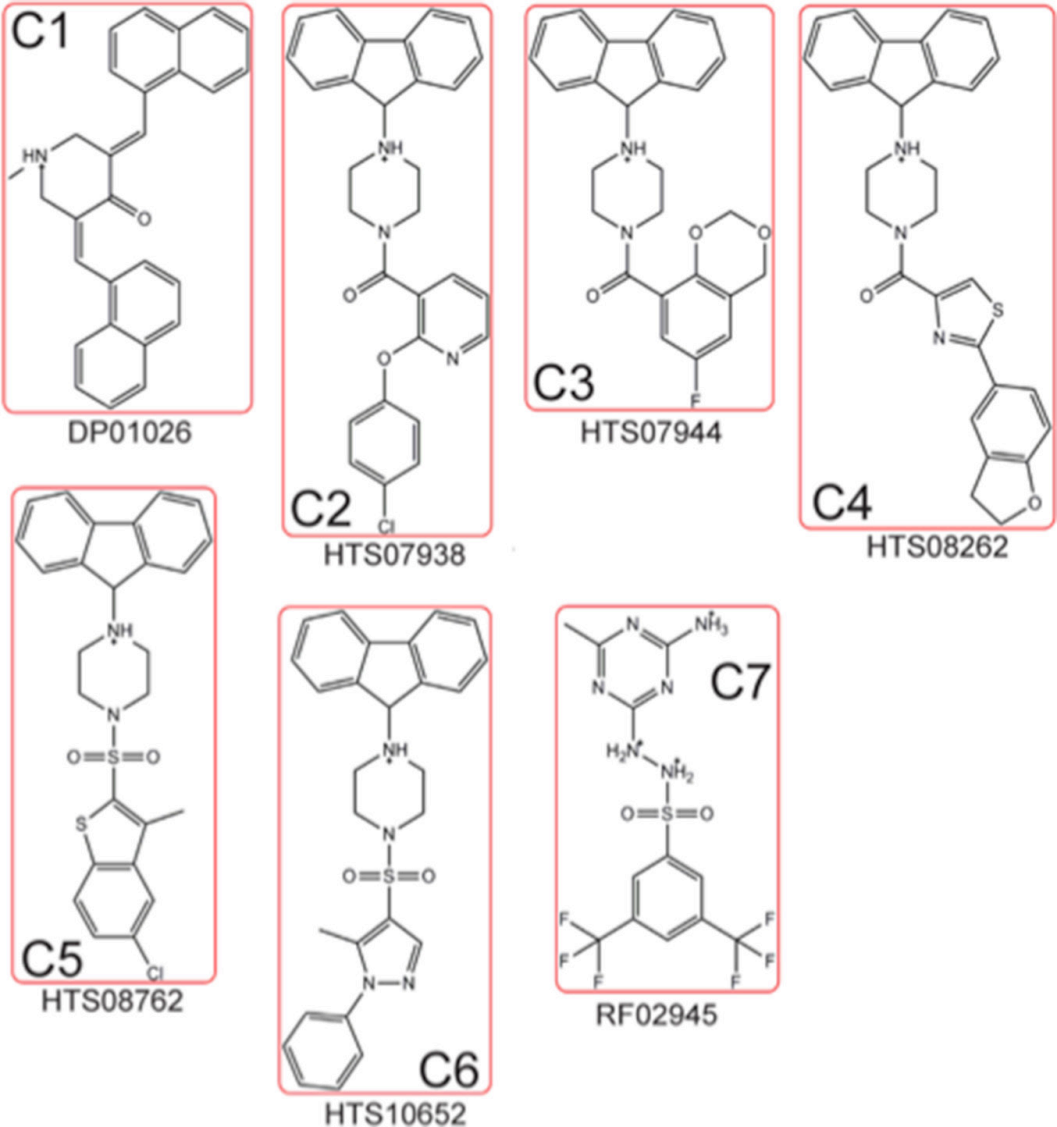
Dock derived compounds tested via *in vitro* screen Dock derived compounds depicted with nicknames (C#) and Maybridge HitFinderTM designations. Structures were generated using ChemDraw.

**Figure 2:**
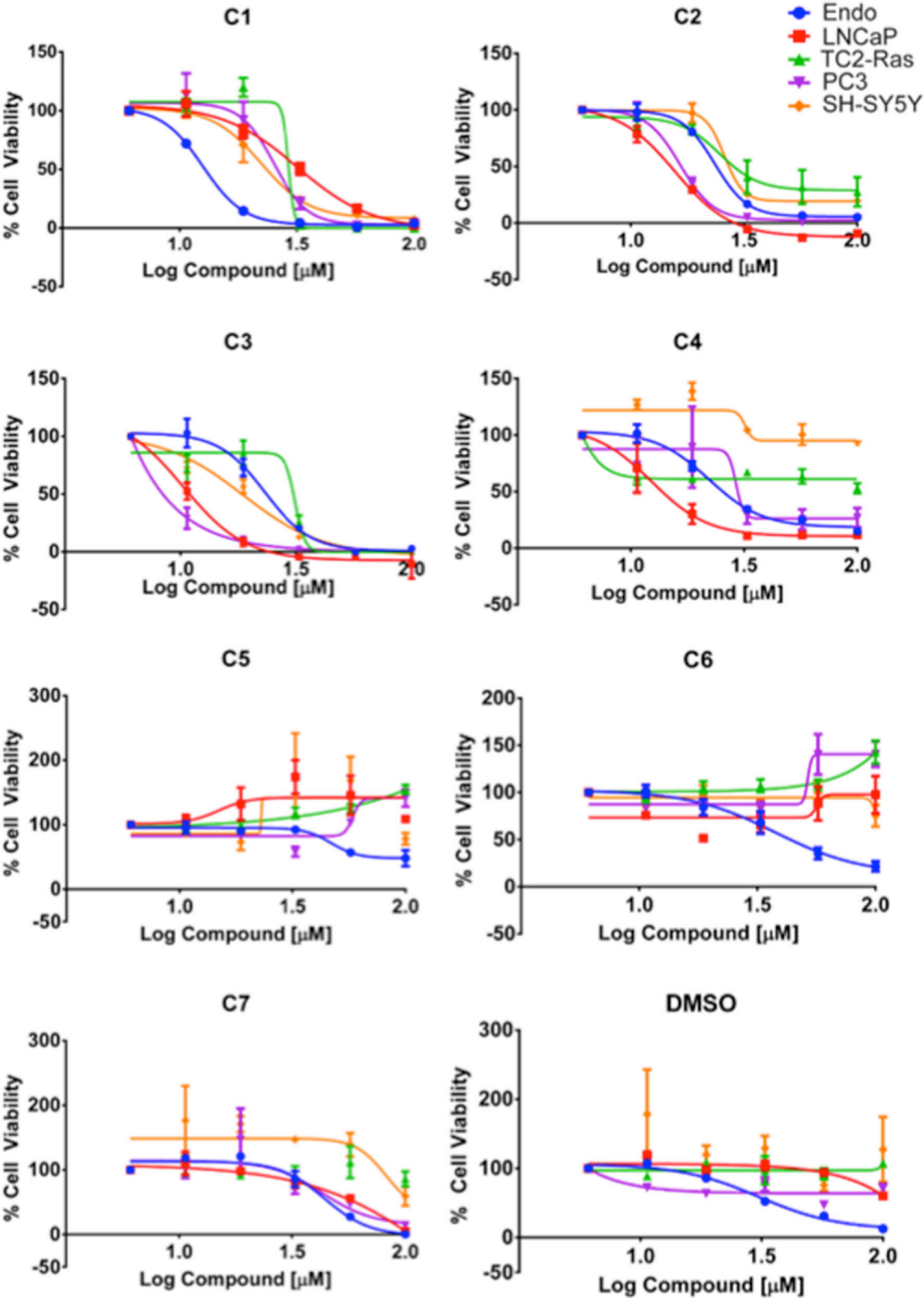
Dose response curves Dose response curves for cell lines treated with 6, 10.5, 18.6, 32.4, 57.5, and 100 µM of compound (log) for 96 hours using % cell viability derived from CCK-8 absorbance readings at 450 nm (*n* = 3, mean ± SEM). A DMSO equivalent control (*n* = 2 ± SEM) was also included for each cell line tested: Ea.hy.926 endothelial cells (Endo), LNCaP androgen dependent metastatic prostate cancer cells, TC2-Ras mouse prostate cancer cells, PC-3 androgen independent metastatic prostate cancer cells, and SH-SY5Y neuroblastoma cells. Curves were fit using GraphPad Prism as described in the methods.

Docking the Maybridge Hitfinder™ library against the 37 LR crystal structure using “Docking @ UTMB” generated 24 compounds with predicted docking scores ranging from –9.3 kcal/mol to –7.9 kcal/mol. After excluding compounds with poor chemical qualities, seven compounds were chosen for further *in vitro* studies. Notably, all compounds contained piperazine-like moieties (Figure 1) and had little to no preexisting data in the chemical literature. We carried out a 96 hour study of cell viability for the initial compound screen because we did not know the relationship between time and dose for the compounds selected *a priori*. A six-dose screen (6 µM to 100 µM) (Figure 2) separated compounds into two groups. C1, C2, C3, and C4 inhibited cell viability in a dose dependent manner whereas C5, C6, and C7 had mixed responses or did not inhibit cell viability in various cell lines. The vehicle, DMSO, had no effect on tumor cell lines LNCaP, TC2-Ras, PC-3, or SH-SY5Y but did have a mild effect on Ea.hy.926 endothelial cells at higher doses. IC_50_s obtained from dose response curves revealed that C3 had the strongest effect on cell viability in the majority of cell lines tested. C3 inhibited cell viability in the androgen dependent LNCaP cell line (10.28 µM), the aggressive and metastatic androgen independent PC-3 cell line (∼0.8 µM), and in the neuroblastoma cell line SH-SY5Y (18.57 µM) (Table 1). Because C3 strongly inhibited cell viability in PC-3 cells, we chose to investigate the mechanism for reduction of cell viability using these cells.

**Table 1:** Tabulated cell viability IC_50s_

96 Hour IC_50_ (µM)	Endo	LNCaP	TC2-Ras	PC3	SH-SY5Y
**C1**	12.54	31.79	∼29.12	25.22	22.19
**C2**	23.44	15.65	24.61	16.52	25.87
**C3**	23.10	10.28	∼31.38	∼0.7577	18.57
**C4**	22.12	12.35	∼5.073	∼29.15	∼31.93
**C5**	47.01	14.84	∼671.5	∼58.42	∼22.94
**C6**	47.01	∼56.36	∼782.7	∼51.66	∼124.0
**C7**	47.01	159.6	—	43.06	82.24

### Effectiveness Of C3 correlates with endogenous 37 LR levels

Because C3 reduced cell viability at lower concentrations in PC-3 and LNCaP cell lines than TC2-Ras, SH-SY5Y, and Ea.hy.926 cell lines, we examined whether C3 IC_50_ values might correlate with endogenous 37 LR levels. PC-3 and LNCaP cell lines expressed 37 LR approximately two fold higher than SH-SY5Y and 1.5 fold higher than Ea.hy.926 and TC2-Ras, with a modest correlation (*r*^2^ = 0.6) between lower C3 IC_50_s and higher expression levels of 37 LR (Supplementary Figure 1).

### Redocking of hit compound C3

**Figure 3:**
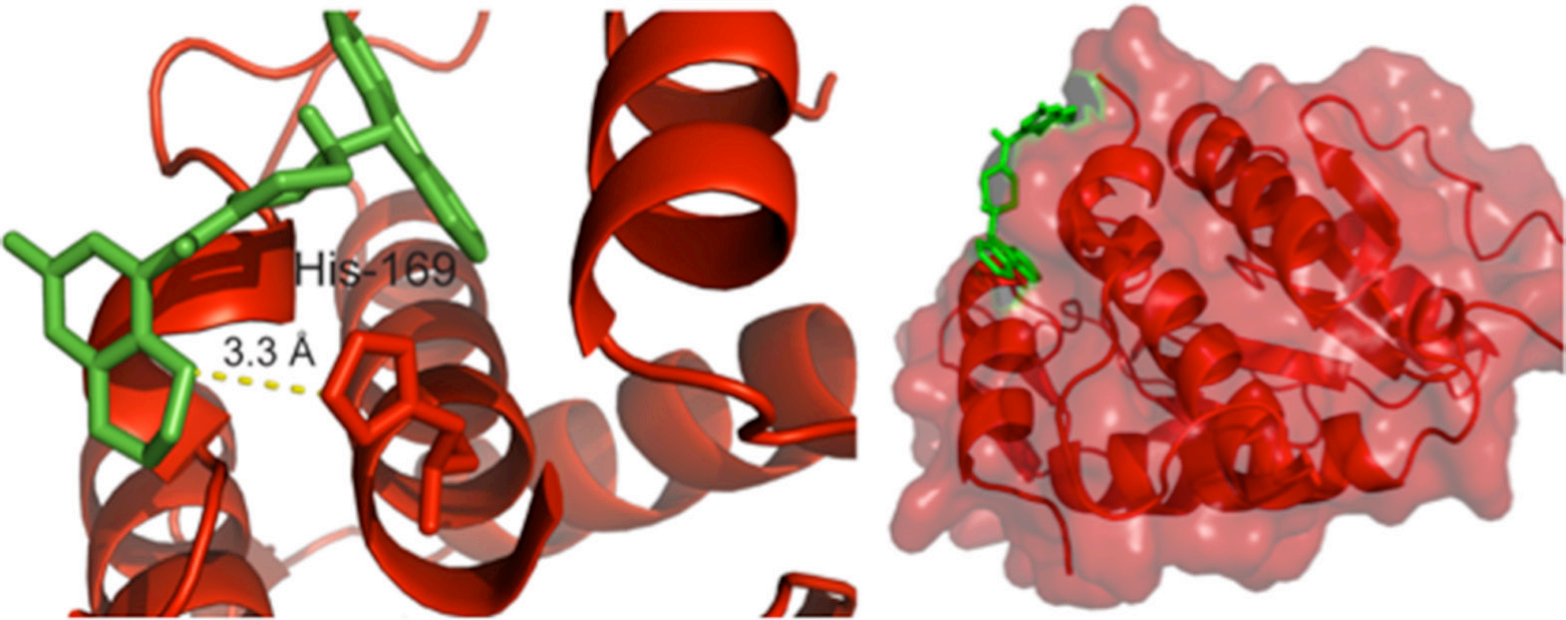
Redocking of hit compound C3 Redocking of C3 was performed as described in the methods and visualized using PyMol. Left, a pose depicting the most energetically favored positioning of C3 from the simulation featuring a 3.3 Å hydrogen bond between the benzodioxin moiety and His 169. Right, the predicted binding pocket for C3 with the 37 LR crystal structure.

C3 was redocked to the 37 LR crystal structure using AutoDock Vina and AutoDockTools to assess predicted binding state and visualized using PyMol. Using a grid encompassing the entire protein as described in the methods, C3 was predicted to interact with His-169 of the 37 LR structure via a 3.3 Å hydrogen bond (Figure 3). Moreover, the predicted interaction site lies within the first binding region for laminin and the known binding region for PEDF. Altering the protonation state of His-169 did not change predicted binding of C3 in simulation (Supplementary Figure 2).

### C3 reduces intrinsic tryptophan fluorescence in peptide G – A peptide representing the 37 LR laminin binding pocket

**Figure 4:**
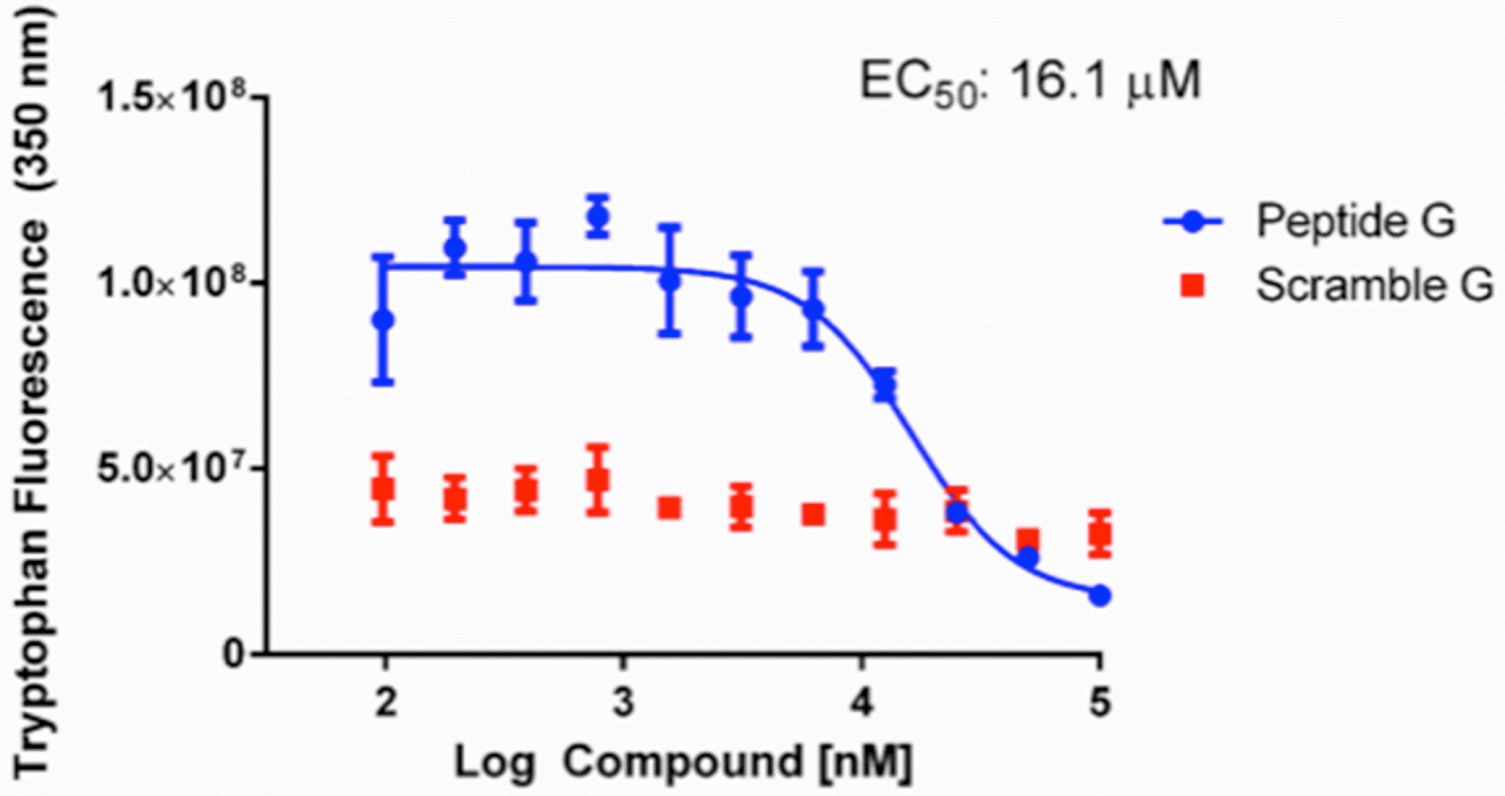
Reduction of tryptophan fluorescence by C3 50 µM Peptide G or Scramble G was incubated with concentrations of C3 ranging from 100 nM to 100 µM. Intrinsic tryptophan fluorescence was quantified at 350 nm emission (270 nm excitation) and graphed as a function of C3 dose after subtracting baseline C3 fluorescence. GraphPad Prism was used to determine an EC50 for tryptophan reduction as described in the methods (*n* = 3 ± SEM).

Computational redocking suggested that C3 engaged 37 LR at His 169, a residue that lies squarely within the laminin binding region of 37 LR and notably, the active laminin binding site spanning amino acids 161 to 180 [61]. After inspecting the amino acid sequence, commonly referred to as Peptide G, we noticed that the sequence contained two tryptophans. We tested the hypothesis that alterations to intrinsic tryptophan fluorescence could be a readout for C3 binding to Peptide G by titrating C3 in an assay buffer containing Peptide G or a 20-mer same sequence scramble (Scramble G) (Figure 4). Titration of C3 from 0.1 to 100 µM reduced intrinsic tryptophan fluorescence in a dose dependent manner with an EC50 of 16.1 µM. Titration of C3 did not appreciably alter the intrinsic tryptophan fluorescence of Scramble G.

### C3 treatment elicits PEDF-like gene expression changes in PC-3 cells

**Figure 5:**
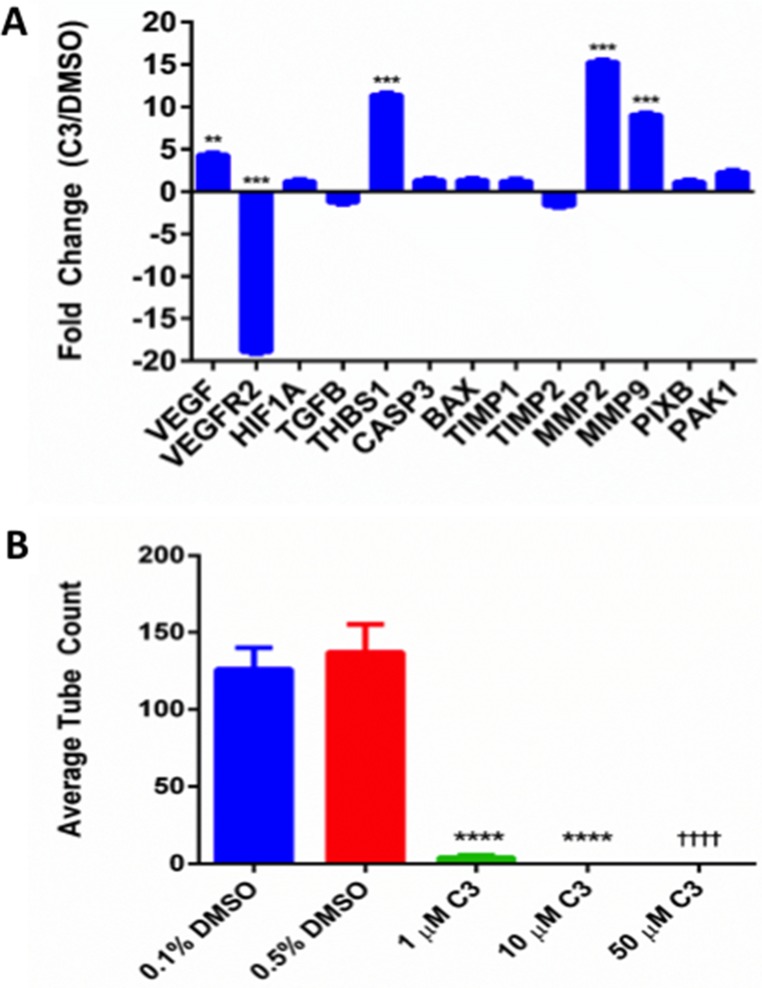
PC-3 response to C3 (A) Expression of PEDF-signaling associated genes from PC-3 cells treated with 50 µM C3, compared to DMSO control and normalized to GAPDH (n=3, mean ± SEM). A Holm-Sidak multiple t test was used for statistical analysis (^*^p < 0.05, ^**^p < 0.01, ^***^p < 0.001). (**B**) Endothelial tube formation assay. Average tube count for 0.1% DMSO, 0.5% DMSO, and 1, 10, and 50 µM treatment with C3 (*n* = 7, mean ± SEM). 1 and 10 µM C3 were compared with respective 0.5% DMSO (unpaired two-tailed t test, ^****^p < 0.0001). 50 µM C3 was compared with respective 0.1% DMSO (unpaired two-tailed t test ^††††^*p* < 0.0001).

Since C3 inhibited cell viability and displayed predicted binding to empirically determined binding sites on 37 LR [34], we used RT-qPCR to screen a panel of known PEDF-signaling related genes involved in PEDF’s ability to both inhibit angiogenesis and activate apoptosis in a cell-type dependent manner (Figure 5A). mRNA levels were examined at 48 hours to provide a window for newly synthesized transcripts to be measured. Genes involved in migration/invasion and/or extracellular matrix (ECM) communication also were assayed. We observed significant (*p* < 0.05) upregulations in vascular endothelial growth factor (VEGF), thrombospondin-1 (THBS1), matrix metalloproteinase (MMP)-2 and -9, and downregulations in vascular endothelial growth factor receptor 2 (VEGFR2). Transforming growth factor beta (TGFB) and tissue inhibitor of metalloproteinase 2 (TIMP2) were downregulated and proapoptotic factors caspase-3 (CASP3) and bcl-2-associated x protein (BAX) and migration/invasion regulators Rho guanine nucleotide exchange factor 7 (PIXB) and PAK1 (P21 (RAC1) Activated Kinase 1) were upregulated as a trend, but these changes were not significant.

### C3 inhibits endothelial tube formation

Because C3 downregulated VEGFR2 and upregulated THBS1 in PC3 cells, we tested the hypothesis that C3 exhibited anti-angiogenic potential using an endothelial tube formation assay (Figure 5B). In this assay, tube formation proceeded uninhibited by the presence of vehicle (DMSO), but not in the presence of 1, 10, or 50 µM of C3 compound (*p* < 0.05). Inhibition of tube formation by C3 did not alter calcein uptake (data not shown), suggesting the inhibition was not driven by a direct apoptotic mechanism.

### C3 does not inhibit cell viability via Caspase 3/7 mediated apoptosis

**Figure 6:**
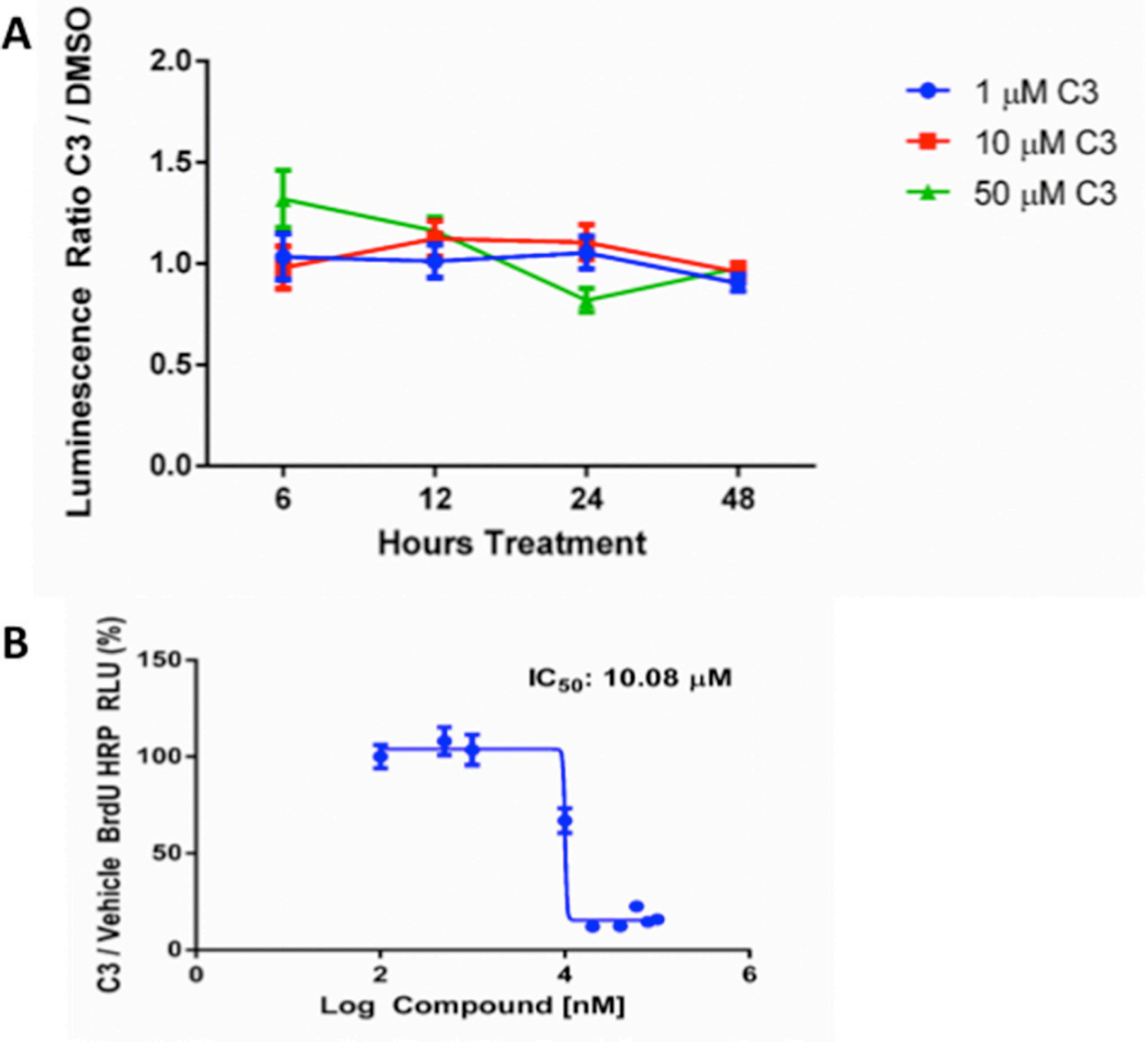
(**A**) **Caspase 3/7 activation in response to C3 treatment**. Average luminescence ratio (active caspase 3/7) for PC-3 cells treated with 1, 10, or 50 µM C3 for 6, 12, 24, and 48 hours (*n* = 3, mean ± SEM) and normalized to DMSO control (results not significant, one-way ANOVA). (**B**) **BrdU Incorporation in response to C3 treatment**. PC-3 cells were treated with 100 nM to 100 µM C3 for 24 hours and BrdU incorporation was quantified using HRP generated luminescence from an antibody pair recognizing BrdU (*n* = 6, mean ± SEM). C3 BrdU signal was normalized to a row where PC-3 cells were exposed to 0.5% DMSO (assay conditions). Curve obtained using GraphPad Prism as described in the methods.

To determine the mechanism for inhibition of cell viability by C3, we treated PC-3 cells with 1, 10, or 50 µM of C3 for 6, 12, 24, or 48 hours continuously and measured activation of caspase 3/7 using a luminescence based assay (Figure 6A). At 6 hours, 50 µM C3 caused a slight increase in caspase 3/7 activity but the results were not significant at any time point for any dose.

### C3 inhibits cell viability via inhibition of cellular proliferation

Since C3 did not appear to inhibit cell viability by an apoptotic mechanism, we tested the hypothesis that PC-3 cells inhibited cell viability due to a reduction in cellular proliferation in response to treatment. A dose curve for C3 was established and bromodeoxyuridine (BrdU) incorporation was used as a readout for proliferation (Figure 6B). We observed inhibition of BrdU incorporation in a dose dependent manner after treatment for 24 hours with an IC_50_ of 10.08 µM.

### Label-free quantitative proteomics suggests C3 alters levels of proteins associated with DNA maintenance, DNA synthesis, and protein translation

**Figure 7:**
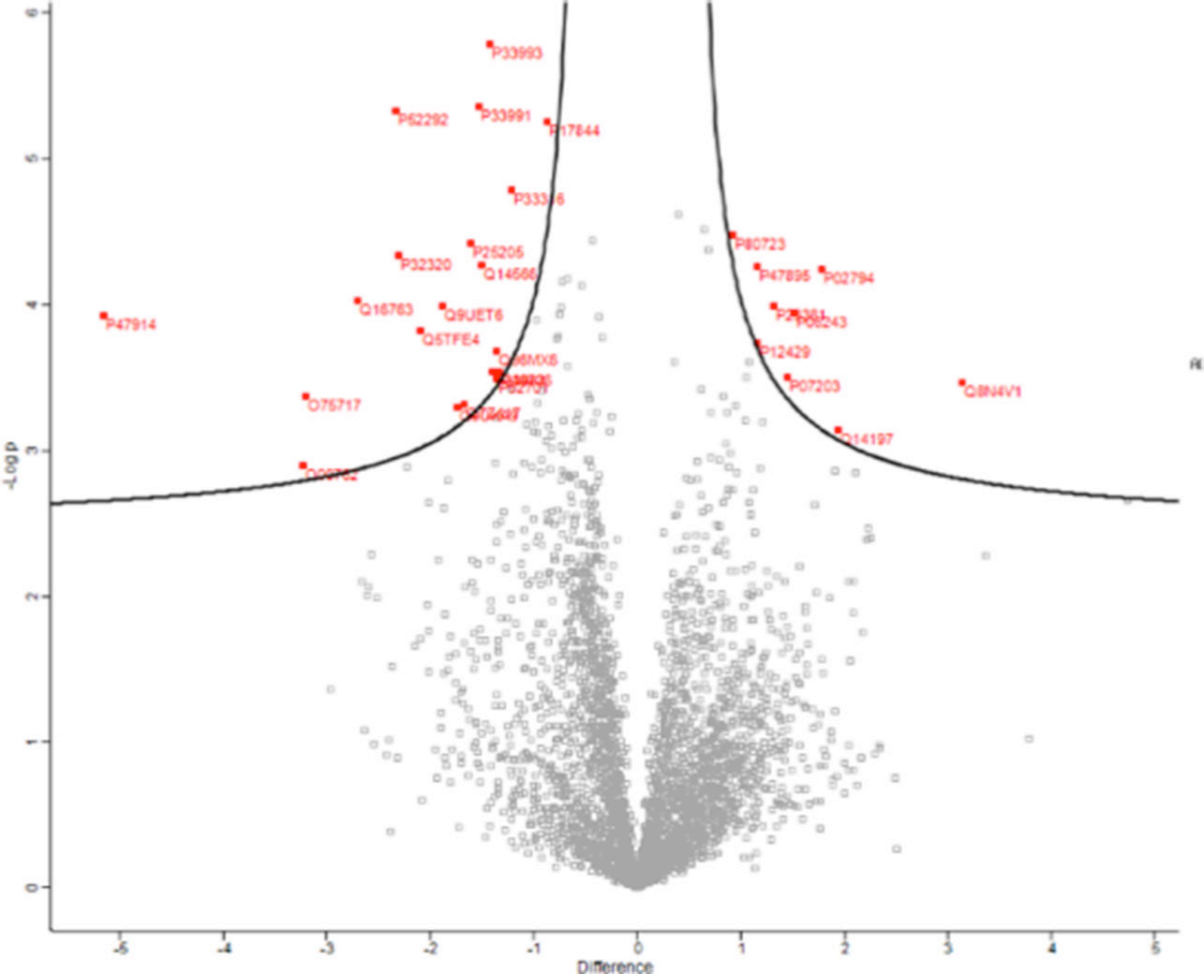
Volcano plot of differentially regulated proteins in response to C3 treatment PC-3 cells were treated with DMSO or 10 µM C3 for 72 hours and proteins were quantified using label free quantitative mass spectrometry. Proteins were graphed by fold change (Difference) and significance (-Log p) using a false discovery rate of 0.05 and an S0 of 0.1. Protein IDs in red were considered significantly up or down regulated using the Perseus software.

To further elucidate potential mechanisms of action for C3, we employed a label free quantitative mass spectrometry approach. Differential changes to the proteome of PC-3 cells were examined at 72 hours to provide a viable window for newly translated proteins to be measured. Perseus was used to generate a volcano plot of significantly up and down regulated proteins from the 3,904 proteins differentially detected between vehicle control (DMSO) and 10 µM C3 after 72 hours of treatment. 29 proteins were significantly differentially regulated in response to C3 treatment (Figure 7) and 16 proteins were significantly differentially regulated at a fold change cutoff of 1.5 (Table 2). Specific protein changes induced by C3 treatment included downregulation of the mini chromosome maintenance (MCM) family of proteins, responsible for replication fork formation. C3 also downregulated two E2 ubiquitin ligases required for mitotic progression (UBE2C and UBE2S). The most downregulated protein (–5.3 FC), RPL29, is incorporated into the 60S ribosome. Additionally, the second most upregulated protein (1.9 FC), ICT1, responds to stalled mitochondrial ribosomes.

**Table 2:** Significantly up and down regulated proteins in response to 10 µM C3

Gene	Uniprot Accession	Protein Name	Log_2_(FC)
***MMGT1***	Q8N4V1	Membrane magnesium transporter 1	3.1
***ICT1***	Q14197	Peptidyl-tRNA hydrolase ICT1, mitochondrial	1.9
***FTH1***	P02794	Ferritin heavy chain	1.8
***ASNS***	P08243	Asparagine synthetase	1.5
***GPX1***	P07203	Glutathione peroxidase 1	1.5
***MCM6***	Q14566	Minichromosome maintenance protein 6	–1.5
***MCM4***	P33991	Minichromosome maintenance protein 4	–1.5
***MCM3***	P25205	Minichromosome maintenance protein 3	–1.6
***NUFIP2***	Q7Z417	Nuclear fragile X mental retardation-interacting protein 2	–1.7
***NT5DC1***	Q5TFE4	5′-nucleotidase domain-containing protein 1	–2.1
***CDA***	P32320	Cytidine deaminase	–2.3
***KPNA2***	P52292	Importin subunit alpha-1	–2.3
***UBE2S***	Q16763	Ubiquitin-conjugating enzyme E2 S	–2.7
***WDHD1***	O75717	WD repeat and HMG-box DNA-binding protein 1	–3.2
***UBE2C***	O00762	Ubiquitin-conjugating enzyme E2 C	–3.2
***RPL29***	P47914	60S ribosomal protein L29	–5.2

### Ingenuity pathway analysis predicts inactivation of Myc, activation of P53, and suggests a doxorubicin like phenotype

**Figure 8:**
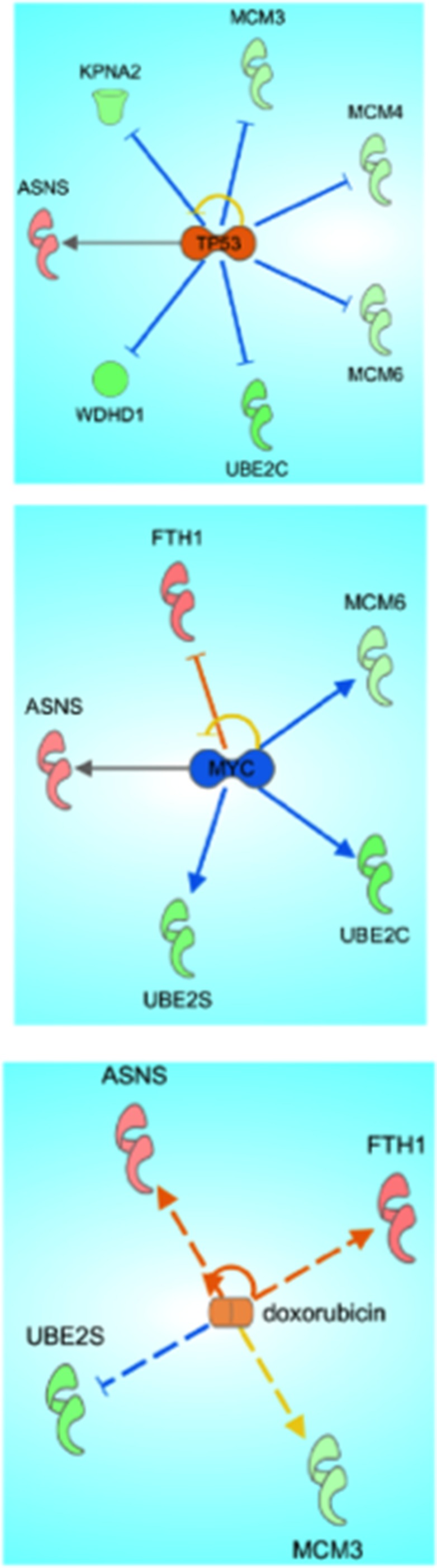
Ingenuity pathway analysis of proteomic data from C3 treatment of PC-3 cells Significantly up and down regulated proteins were analyzed using Ingenuity Pathway Analysis Software and mapped to canonical pathways.

Proteomics analyses utilizing Ingenuity Pathway Analysis (IPA) of significant (*p* < 0.05, FC 1.5) proteins altered upon treatment with 10 µM of compound C3 suggested a predicted activation of tumor suppressor p53 pathways and a predicted deactivation of tumor promoter c-myc (Figure 8). IPA predicted an activation of P53 corresponding to the experimentally detected downregulations of MCM family proteins and other proteins involved in DNA synthesis and integrity (KPNA2, UBE2C, WDHD1). IPA predicted an inactivation of c-myc corresponding to downregulation of MCM6, UBE2C, and UBE2S and the upregulation of ASNS (asparagine synthase). The proteomic signature obtained with C3 compared to vehicle control (DMSO) was linked to the well-characterized chemotherapeutic doxorubicin by IPA.

### C3 inhibits c-Myc binding activity at 50 µM

**Figure 9:**
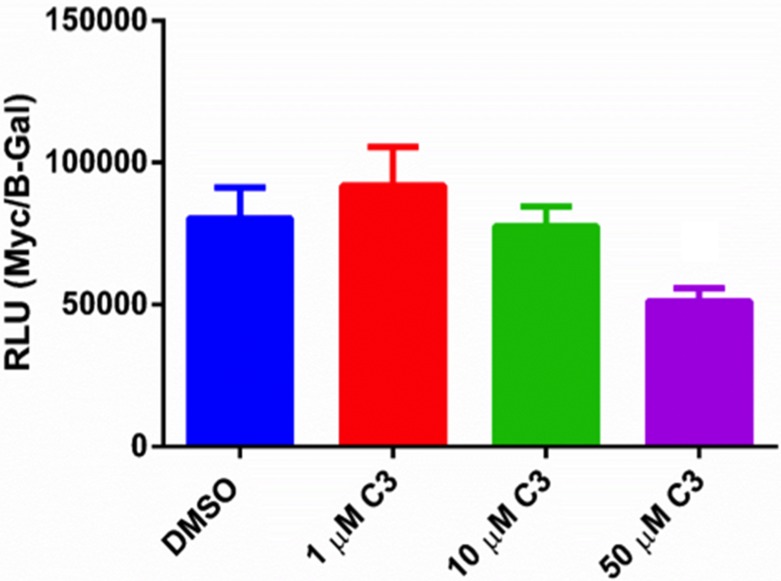
Luciferase promoter assay in PC-3 cells PC-3 cells were treated with 1, 10, 50 µM C3 or DMSO control for 24 hours after a 24 transfection with Myc-Luc and a B-gal plasmid to normalize for transfection efficiency (*n* = 6, mean ± SEM). Relative light units calculated by normalizing Myc-Luc signal to B-gal signal. A one-way ANOVA was used for statistical analysis (*p* < 0.05). The linear trend was also significant (post test for linear trend, *p* < 0.05).

Because P53 status and function is impaired in PC-3 cells, we chose to orthogonally validate the IPA prediction of c-myc inactivation using a promoter-based reporter assay (Figure 9) consisting of four c-myc binding sites upstream of a luciferase gene [62]. 1 and 10 µM C3 had no significant effect on luciferase reporter activity however 50 µM C3 significantly reduced c-myc binding activity by 36%.

### Compound C3 inhibits wound healing in a scratch assay using PC3 cells

**Figure 10:**
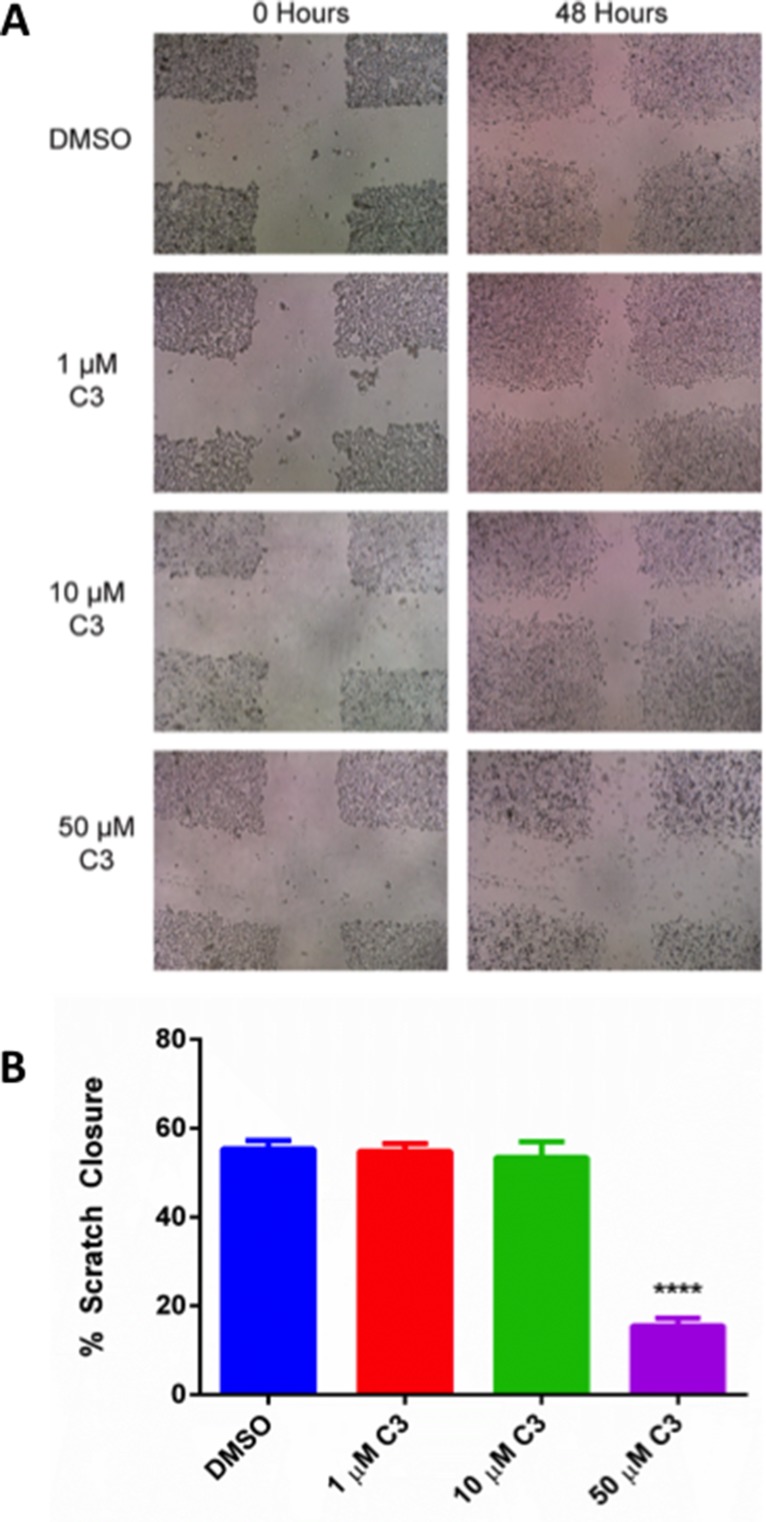
(**A**) **Representative Scratch Assay brightfield images for PC-3 cells in response to C3 treatment**. PC-3 cells were treated with 1, 10, 50 µM C3 or DMSO control after scratch formation and images were recorded using a brightfield microscope as described in the methods. (**B**) **Quantification of Scratch Assay**. Brightfield images were quantified using the t-scratch program and represented as % scratch closure after 48 hours of migration compared to a 0 hour control (*n* = 9, mean ± SEM). A one-way ANOVA test was used for statistical analysis (*p* < 0.05). A post-hoc Sidak’s multiple comparisons test was also used (^****^p < 0.0001, 50 µM C3 vs. DMSO, all other doses vs. DMSO were not significant).

Because C3 inhibited growth pathways in several different assays, we decided to investigate whether C3 could inhibit wound healing in a scratch test assay as a measure of cancer cell aggressiveness as it relates to their migration capacity. The scratch assay is a well-developed method for measuring cell migration *in vitro* and was chosen because it mimics cell migration during wound healing *in vivo* and is compatible with live cell imaging [63]. In this assay, we utilized the t-scratch method to assess the ability of compound C3 to promote wound closure or healing. PC3 cells were treated for 48 h with vehicle control (DMSO), or 1, 10, or 50 µM compound C3. A t-scratch was made in the center of the wells and imaged over time using a brightfield microscope (Figure 10A).Quantification of the wound closure was performed as described in materials and methods using tscratch software. We observed that compound C3 prevented wound closure by ∼70% at 50 uM (*p* < 0.05; Figure 10B).

## DISCUSSION

We have demonstrated that phenotypic cell survival assays can be effectively coupled to virtual based screening methods to identify novel compounds that alter and inhibit castration resistant prostate cancer cell (PC-3) growth in the context of the PEDF/LR interaction interface. It has been shown that *in silico* approaches to drug discovery are a useful tool for cancer biologists and facilitate hypothesis driven research avenues without the expensive startup costs associated with biochemical drug discovery that may be underutilized on compounds that do not display desirable anti-tumor properties in downstream *in vitro* and *in vivo* functional assays [64]. Moreover, *in silico* approaches are becoming more accessible [65] and user friendly and the datasets generated can be explored and readily refined with input from expert medicinal chemists.

HTS07944, or C3, was able to inhibit cell viability at ∼1 µM and inhibited cellular proliferation at 10 µM in PC3 cells. Additionally, a 50 µM dose was sufficient to inhibit cell migration in a wound healing assay. Taken together, C3 had a clear effect on PC-3 cell viability and function. C3 is a piperazine analogue, a class of compounds well characterized for their diverse uses in medicinal chemistry ranging from neuropsychiatric applications [66], tuberculosis treatment [67], anti-angiogenic therapy [68], to cancer [69]. Our *in silico* structural predictions suggest that C3 binds to 37 LR in the laminin binding pocket and that this binding may be facilitated by a hydrogen bond between His 169 and the benzodioxin moiety of the compound. This hypothesis is supported by recent work highlighting a class of compounds from (or derived from) the NCI Diversity Set that were predicted to interact with the same general binding pocket, termed peptide G, of 37 LR (161 to 180 aa) [60], albeit through hydrogen bonding with Gly 172 and a hydrophobic interaction with His 169. Of relevance, this binding region falls squarely within the known interaction region for PEDF and 37 LR (120 to 210 aa) [34]. Our *in silico* predictions were validated by an intrinsic tryptophan fluorescence assay employing Peptide G (161 to 180 aa), whereby titration of C3 decreased intrinsic tryptophan fluorescence in a dose dependent manner with an EC50 of 16.1 µM while having no appreciable effect on tryptophan fluorescence in a same sequence scrambled peptide (Scramble G), suggesting that C3 binds specifically to the intrinsic fold of Peptide G. Taken together with the evidence that C3’s potency (reduction in cell viability) is correlated with endogenous 37 LR levels, we suggest that C3 interacts with 37 LR.

Our lab and others have demonstrated that PEDF’s potent antiangiogenic potential can be leveraged to treat both the tumor and the surrounding angiogenic microenvironment [39, 46–50]. While these gene delivery driven therapies show promise, we sought to identify small molecule that could recapitulate known signaling properties of PEDF but without the additional complications of peptide formulation, delivery of a recombinant protein, or gene delivery safety validation. In our hands, we were unable to demonstrate a significant dose/response relationship between survival of PC-3 cells and increasing doses of recombinant PEDF (Supplementary Figure 3). This was not surprising to us as the anti-cancer/anti-angiogenic PEDF peptide p18 does not markedly alter PC-3 cell viability while still maintaining an *in vivo* potency [39], however, the result could also be due to variability inherent to PEDF preparations [70].

We investigated the expression of a panel of genes implicated in either proposed mechanisms for PEDF function or for 37 LR function. Treatment of PC-3 cells with 50 µM C3 for 48 hours markedly reduced transcription of VEGFR2 (> –15 fold change or FC), a known effect of PEDF treatment, previously shown to be mediated through a gamma secretase dependent proteolysis of VEGFR2 [71]. A compensatory upregulation of VEGF was observed (∼4 FC) but with no concomitant upregulation of HIF1A. The known upregulation of MMP2 and MMP9 in response to PEDF treatment was also observed. However, where recombinant or endogenous full length PEDF protein is a substrate for proteolytic degradation [71] by MMP2 or MMP9, a small molecule that mimics PEDF should avoid such degradation, as the specific proteolytic sites are not present in C3. C3 also upregulated thrombospondin-1 (THBS1, >10 FC). THBS1, much like PEDF, has been implicated as a protein with dual anti-tumor and anti-angiogenic activity [72]. Additionally, the two proteins work in concert to downregulate angiogenesis [41]. Consistent with our finding that C3 did not alter cell viability via a caspase 3/7 dependent mechanism, we did not observe a significant upregulation of caspase 3 (CASP3) or BAX at the mRNA level. Overall, these results suggest that C3 could mimic PEDF responsive-genes or pathways in a manner consistent with an anti-angiogenic function.

While VEGF receptors have been demonstrated to be expressed by PC-3 cells and to facilitate an autocrine VEGF mediated signaling loop that may promote tumor growth [73], we wanted to orthogonally validate the C3-induced PEDF associated gene changes by testing the compound in an endothelial cell tube formation assay. 1, 10, and 50 µM doses of C3 were able to completely or nearly completely inhibit endothelial tube formation, suggesting that C3’s observed effects on angiogenesis-associated genes correlates with functional inhibition of angiogenesis.

Because the functions of 37 LR and PEDF are diverse and the mechanisms underlying PEDF’s anti-tumor properties are still unclear (in contrast with its well-characterized role in anti-angiogenesis), we employed an unbiased label-free quantitative mass spectrometry approach to determine the types and levels of proteins modulated in PC-3 cells following treatment with a moderate dose of C3 (10 µM), chosen from the BrdU and t-scratch functional assays. Our objective was to gain insight into the potential mechanisms of activity of C3 in PC-3 cells through proteomic analyses. The most downregulated protein (RPL29, log2FC –5.2), is a member of the 60S ribosome which indicates that C3 may be acting to modify translational pathways, consistent with a hypothesis that C3 is exerting anti-tumor effects through 37 LR. Moreover, RPL29 binds and interacts with the extracellular matrix and knockdown of RPL29 has been reported to induce colon cancer cell differentiation [74]. siRNA knockdown of RPL29 in an aortic model of angiogenesis resulted in significantly decreased microvessel sprouting in response to VEGF stimulation [75]. These alterations might suggest a role for C3 in the regulation of angiogenesis through impacting regulation of translation.

Consistent with our observations that C3 inhibited cell viability, cell proliferation, and cell migration, we observed a downregulation of several MCM family proteins, MCM-3, MCM-4, and MCM-6. MCM proteins serve as a checkpoint for the S phase of the cell cycle and are considered diagnostic markers for poor prognosis in a variety of cancers [76–78]. Further evidence for disruption of DNA synthesis, as evidenced by C3′s ability to inhibit BrdU incorporation, was confirmed by down regulation of the E2 ligases UBE2S and UBE2C, proteins required for mitotic exit [79, 80]. UBE2C is considered a potent oncogene and is highly upregulated in castration-resistant prostate cancer [81]. The mitotic/centromere associated protein WDHD1 was also downregulated [82]. C3 decreased levels of CDA, cytidine deaminase, an enzyme well characterized for its ability to decrease the efficacy of gemcitabine chemotherapy [83], suggesting that C3 could be explored in the future as a part of a combinatorial chemotherapeutic regimen. We also observed downregulation of KPNA2, an importin and a key marker of poor disease prognosis and metastatic prostate cancer aggressiveness [84]. In combination, these changes suggest a role for C3 in regulating cancer cell aggressiveness through regulation of mitotic/cell division machinery function.

C3 also upregulated GPX1, glutathione peroxidase 1, a response protein that facilitates sequestration of cytotoxic oxidants and a described putative tumor suppressor with loss of function in PC-3 cells [85]. Upregulation of FTH1, ferritin heavy chain 1, may be a compensatory response to C3’s effects on angiogenic pathways, as FTH1 can reestablish tumor angiogenesis [86]. Upregulation of ASNS, asparagine synthetase, may also be a compensatory response, as ASNS is upregulated under cellular stress conditions including the unfolded protein response or endoplasmic reticulum stress [87]. Consistent with the hypothesis that C3 may disrupt translation events, we noted an upregulation of ICT1, a peptidyl tRNA hydrolase that responds to mitoribosomal arrest [88], but the significance of this finding is unknown since ICT1 may be implicated in cancer progression [89].

While the majority of the proteins detected via mass spectrometry appeared to support the hypothesis that the net effect of C3 on PC-3 cells was an activation of anti-tumor signaling, we further analyzed the dataset using ingenuity pathway analysis to assess the effects of C3 *en masse*. Pathway analysis indicated a *predicted* activation of P53, a *predicted* inactivation of MYC, and suggested that the signature of proteins altered by C3 was consistent with the known effects of doxorubicin. Both activation of P53 and inactivation of MYC involved the MCM family of proteins detected in this study as well as UBE2S and/or UBE2C. We chose to orthogonally validate our proteomic data set and IPA interpretations using a luciferase reporter system for MYC binding elements, reasoning that PC-3 cells are functionally considered P53 null [90] and that advanced prostate cancers frequently overexpress c-myc [91]. 50 µM C3 was able to significantly decrease c-myc binding activity in a reporter assay, validating our proteomic results.

Taken together, we propose that C3’s phenotypic effects on PC-3 cells are a dual combination of anti-tumor/anti-growth pathway and anti-angiogenic pathway activation, of which there is some overlap. The dual anti-tumor/anti-angiogenesis activity is consistent with the described effects of PEDF reported by us and others. Given that C3 inhibits BrdU uptake, downregulates MCM family proteins, and downregulates c-myc activity, we propose that C3 is a *bona fide* anti-proliferative agent that warrants further pre-clinical investigation in the realm of prostate cancer drug discovery and development, and that 37 LR continues to be a promising avenue for targeted therapeutic intervention. We believe future efforts in the 37 LR targeting field would benefit from development of a high throughput binding assay and we aim to test both C3 and derivatives of C3 in such a format, perhaps employing a tryptophan fluorescence assay using Peptide G. A peptide based high-throughput binding assay is economical and facilitates true structure activity relationships to be derived between Peptide G/37 LR and lead iterations of C3 or other 37 LR active compounds. Moreover, we plan to test the efficacy of C3 in a mouse model of prostate cancer prior to lead optimization.

## MATERIALS AND METHODS

### *In silico* screening ligand & protein preparation

The 2.15 *Å* crystal structure of 37 LR (PDB ID: 3BCH) [92] was cleaned and prepared using AutoDock Vina and AutodockTools [93]. The Maybridge HitFinder™ version 10 library comprising 14,400 drug-like compounds was accessed using “Docking At UTMB” (now “Docking At TACC”), a virtual screening drug discovery web portal that performs automated docking of pre-cleaned .pdbqt libraries against an input structure [65]. The top 24 compounds (–9.3 kcal/mol to –7.9 kcal/mol) were recorded and evaluated for downstream applications.

### Refinement of *in silico* hits

The top 24 compounds as assessed by predicted binding scores via docking were evaluated for drug-likeness by a medicinal chemist. 7 final compounds were ordered and synthesized from Maybridge via ThermoFisher Scientific. Compounds were resuspended in 100% dimethylsulfoxide (DMSO, Sigma Aldrich) to 10 mM, vigorously mixed, inspected for absence of precipitates, and stored at –20°C in amber tubes until use. Maybridge compound names, clogp, and nicknames are provided in Table 3.

**Table 3:** Selected compounds

Compound	Nickname	cLogP	MW (Da.)
**DP01026**	**C1**	6.1	389.496
**HTS07938**	**C2**	5.18	481.981
**HTS07944**	**C3**	4.53	430.477
**HTS08262**	**C4**	4.06	430.477
**HTS08762**	**C5**	6.1	430.477
**HTS10652**	**C6**	4.54	470.594
**RF02945**	**C7**	2.99	416.301

### Cell culture conditions

All cell lines were routinely passaged and maintained at 37°C with 5% CO_2_. EA.hy926 endothelial cells (Endo, ATCC CRL-2922) were cultured in 10% fetal bovine serum (FBS, ThermoFisher) with 1× Dulbecco’s Modified Eagle Medium (DMEM, Corning) and 1× antibiotic-antimycotic (anti-anti, 100 units/mL penicillin, 100 µg/mL streptomycin, and 0.25 µg/mL amphotericin B; Gibco). LNCaP prostate tumor cells (ATCC CRL-1740) were cultured in 10% FBS (ATCC) with 1X RPMI-1640 (Corning) and 1× anti-anti. PC-3 prostate tumor cells (ATCC CRL-1435) were cultured in 10% FBS (ThermoFisher) with 1× RPMI-1640 and 1× anti-anti. SH-SY5Y neuroblastoma cells (gift from Dr. Rakez Kayed) were cultured in 10% FBS (ThermoFisher) with 1X Opti-MEM I (Gibco) and 1× anti-anti. Mouse TRAMP-C2 cells were obtained from ATCC and maintained in Dulbecco’s Modified Eagle’s Medium Nutrient Mixture F-12 (DMEM:F12; Corning) with 10% FBS (ThermoFisher) and 1× anti-anti. TRAMP-C2 cells [94] were transduced with a lentivirus expressing activated H-rasG12V (Lv-Hras) [95] at a multiplicity of infection of 1 and a lentivirus expressing mouse androgen receptor (mAR) at a multiplicity of infection of 1, resulting in TC2-Ras. Their characterization is shown in Supplementary Figure 4. Cells were regularly passaged by trypsinization (0.05% (v/v) trypsin, 0.53 mM EDTA) or lifted using 20 mM EDTA (Sigma) in 1X DPBS.

### Cell viability screen

PC-3, Endo, TC2-Ras, SH-SY5Y, and LNCaP cells were seeded in 96-well plates at 1,500 cells per well using the Scepter cell counter (EMD Millipore). Cells were continuously incubated with compound using a 6-point dose curve: 100, 57.5, 32.4, 18.6, 10.5, and 6.0 µM in their respective complete media. Cell viability was measured by using the cell counting kit-8 (CCK-8, Dojindo). CCK-8 uses a highly water soluble tetrazolium salt, WST-8, which is reduced by dehydrogenase in cells to yield a yellow formazan dye, which is directly proportional to the number of living cells. CCK-8 absorbance was measured at 450 nm using a GloMax Multimode Reader (Promega). Readings were performed the day after seeding to normalize for variations in cell seeding and after 96 hours of compound treatment. After each reading, CCK-8 media was removed and replaced with media containing compound as described previously. A serial dilution using vehicle, dimethyl sulfoxide (DMSO), for each cell line was performed in parallel along with inclusion of a media only negative control and a cell only positive control.

### Cell viability screen statistics

As described previously, an initial baseline CCK-8 reading was performed at 24 hours post seeding to correct for variations in seeding density. First, 450 nm values for each well were subtracted from media only readings to correct for baseline absorbance. Then, the 96 hour readings were normalized to their respective 24 hour readings to control for seeding variation. Percent cell viability was obtained by dividing corrected 96 hour values by untreated cell only control readings, which were normalized to 100% on the y-axis. Average compound values were reported in triplicate and standard error of the mean (SEM) calculated per plate, with DMSO values reported in duplicate with SEM per plate. Since the compounds were designed to mimic PEDF, an inhibitor of 37 LR, we postulated that we could calculate IC_50_ values for each compound. IC_50_ represent the concentration of an inhibitor where the response is reduced by half. IC_50_s were calculated using a nonlinear fit (log(inhibitor) vs. response – variable slope (four parameters)) in GraphPad Prism 6.

### Endogenous 37 LR expression levels in screened cell lines

PC-3, Endo, TC2-Ras, SH-SY5Y, and LNCAP cells were grown in 100 mm plates in complete media and lifted at ∼90% confluency using 20 mM EDTA in 1× DPBS. Cells were pelleted, media was aspirated, and pellets were frozen at –80°C until required for analysis. Total protein was isolated using 1X RIPA buffer (Thermo Scientific) containing 1X Halt Protease Inhibitor (Thermo Scientific), 5 mM EDTA, and Phosphatase Inhibitor Cocktails 2 and 3 (Sigma Aldrich). Cells were lysed passively on ice for thirty minutes, sheared using a tuberculin syringe (BD) and supernatant was separated from cellular debris after a 30 minute 14,000 rpm spin at 4°C. Protein concentration was determined using the BCA assay (Thermo Scientific). 50 µg of protein was loaded onto a Bolt 4–12% Bis-Tris Plus gel (Life Technologies) and electrophoresed for 30 minutes at 200V. Proteins were transferred onto nitrocellulose using the iBlot2 system (Life Technologies). The blot was blocked for 1 hour at room temperature using 5% BSA in 1X PBS with 0.1% Tween-20 (Acros). 37 LR was probed using a rabbit polyclonal (Bioss, bs-0900R, 1:250) and beta actin was probed using a mouse monoclonal (ThermoFisher, MA5-15739, 1:5000). Goat anti-rabbit IgG secondary (Licor, 925-68021, 1:15,000) and goat anti-mouse IgG secondary (Licor, 925-32210, 1:15,000) were used to detect primary antibody and signal was detected using the Licor Odyssey CLx. Image Studio Lite (Licor, ver. 5.2) was used to quantify pixel intensity.

### Redocking and structural validation of hit compound C3

Redocking of C3 was manually performed using Autodock Vina and AutodockTools with a 100 Å box with a center of (13.8, 52.9, 39.2 (x,y,z)) and an exhaustiveness of 1000. Pymol was used for visualization. C3 structure was confirmed using 1H-NMR, 13C-NMR, and HPLC-MS-MS (data not shown).

### Intrinsic tryptophan fluorescence binding assay

A 20-mer peptide encompassing the 37 LR binding site for laminin (aa 161–180) [61] was synthesized alongside a same-sequence 20-mer scramble peptide: IPCNNKGAHSVGLMWWMLAR (Peptide G), GGKMLWISVANNRLCMAPWH (Scramble G), at 98% percent purity (United Biosystems). Dry peptides (∼5 mg) were resuspended in 50 µL of DMSO and diluted dropwise with 450 µL of dH_2_O. Final peptide concentration was determined via A280 using a Nanodrop by accounting for the molar absorptivity of the two tryptophans present in the peptides. For the binding assay, C3 was serially diluted from 100 µM to 0.1 µM in assay buffer (50 µM Peptide G or Scramble G, 1% DMSO and 1× PBS) or in assay buffer containing no peptide using a 96 well black plate (BD Falcon). Using a SpectraMax i3 MultiMode instrument (Molecular Devices), spectra were collected in 1 nm increments from 300 nm to 460 nm using an excitation wavelength of 270 nm. Tryptophan quenching was graphed as change in 350 nm fluorescence as a function of C3 concentration after subtracting baseline C3 fluorescence.

### BrdU incorporation assay

PC-3 cells were seeded overnight in a white reflective 96 well plate (Costar) at 1,500 cells per well. Media was aspirated and cells were treated with 100 nM, 500 nM, 1 µM, 10 µM, 20 µM, 40 µM, 60 µM, 80 µM, or 100 µM C3 diluted in the previously described media with the addition of 0.5% DMSO. Compounds were incubated for 24 hours at 37°C with 5% CO2 and assayed for BrdU incorporation using the manufacturer’s kit and protocol (Cell Signaling Technology). Relative light units were quantified from Brd-U HRP using a GloMax MultiMode Reader. C3 signal was normalized to PC-3 cells exposed only to media with 0.5% DMSO. An IC_50_ was calculated as described.

### Apoptosis assay

PC-3 cells were seeded overnight in white reflective 96 well plates at 15,000 cells per well. Media was aspirated and cells were treated for 6, 12, 24, or 48 hours with 1 µM, 10 µM, or 50 µM C3 diluted in the previously described media with the addition of 0.5% DMSO. Media was aspirated and cells were assayed for caspase 3/7 activity using a CaspaseGlo kit (Promega). Relative luciferase units were quantified using a GloMax MultiMode Reader.

### RT-qPCR

PC-3 cells were seeded in 6 well plates at 3 × 10^5^ cells per well and incubated with 50 µM C3 or DMSO equivalent in 1× RPMI-1640, 1X anti-anti, and 2% FBS (ThermoFisher) for 48 hours. PC-3 cells were lifted using 20 mM EDTA in 1X DPBS and pelleted before isolating total RNA using the RNeasy Mini Kit (Qiagen). 1 µg of RNA per sample was reverse transcribed using the amfiRivert cDNA synthesis master mix (GenDEPOT). Real-time PCR wells contained 1 µL cDNA template, 2× SYBR Green Master Mix (Applied Biosystems), and 20 µM forward and reverse primers. Primer sequences are provided in Table 4. qRT-PCR was performed on an Eppendorf Realplex 2S (Eppendorf), using: 40 × 95°C for 3 mins; 95°C for 3 s; 60°C for 30 s; 72°C for 8 s and analyzed using EP Realplex software (version 2.2). Fold changes were normalized to GAPDH using the ddCT method.

**Table 4:** List of primers

Gene	Forward Primer (5′–3′)	Reverse Primer (5′–3′)
*VEGF*	CACTGAGGAGTCCAACATCACC	CTGCATTCACATTTGTTGTGC
*VEGFR2*	CCAGTCAGAGACCCACGTTT	AGTCTTTGCCATCCTGCTGA
*HIF1A*	AGTCAAGGGCATATCCTACAACA	CCTTATCAAGATGCGAACTCACA
*TGFB*	CCCGGGTTATGCTGGTTGA	AAGGACCTCGGCTGGAAGTG
*THBS1*	TGCTATCACAACGGAGTTCAGT	GCAGGACACCTTTTTGCAGATG
*CASP3*	ATGGAAGCGAATCAATGGAC	GGCTCAGAAGCACACAAACA
*BAX*	TCATGAAGACAGGGGCCTTTT	CAATCATCCTCTGCAGCTCCA
*TIMP1*	ACCACCTTATACCAGCGTTATGA	GGTGTAGACGAACCGGATGTC
*TIMP2*	GCTGCGAGTGCAAGATCAC	TGGTGCCCGTTGATGTTCTTC
*MMP2*	GTGCAGCTCTCATATTTGCCC	AAGAAGTATGGCTTCTGCCCTGAG
*MMP9*	GGATGCCATTCACGTCGTCCTTAT	TGGGCTTAGATCATTCCTCAGTGC
*PIXB*	GTGTACCTCACGTATTGTGCC	CCGTGGTCAGCACGAGAAT
*PAK1*	CAACTCGGGACGTGGCTAC	CAGTATTCCGGGTCAAAGCAT
*GAPDH*	ACAACTTTGGTATCGTGGAAGG	GCCATCACGCCACAGTTTC

### Wound healing assay

PC-3 cells were seeded in 6 well plates at 3 × 10^5^ cells per well in complete media until the cells reached confluency. Three horizontal and three vertical scratches were made in the confluent monolayers using a standard 200 µL pipette tip and cellular debris were removed by washing with 1× DPBS three times. 1× RPMI-1640 supplemented with C3 or DMSO was added and cells were incubated for 48 hours as described previously. 0 hour and 48 hour images were acquired using a bright field microscope (VWR) and repositioning at 48 hours was achieved using x,y stage coordinates after washing the monolayer three times with 1× DPBS to remove debris. Images were uploaded and analyzed for scratch geometry using the tscratch program [96]. First, global thresholding was performed, then individual images were manually inspected for single image threshold adjustment and scratch geometries were quantified.

### C3 dose experiment for proteomics

PC-3 cells were seeded at 2.5 × 10^5^ cells per well in six well plates in complete media and treated with 10 µM C3 or 0.1% DMSO for 72 hours with a media change (also containing compound or vehicle) at the 48 hour mark. After 72 hours, cells were lifted using 20 mM EDTA in 1X DPBS, pelleted, snap frozen in liquid nitrogen and stored at –80°C until digested for mass spectrometry. A moderate 10 µM dose was chosen from the BrdU and t-scratch functional assays.

### Sample digestion

Prior to digestion, cells were lysed using the Barocycler NEP2320 (Pressure Biosciences, Inc.). 50 µl of 100 mM ammonium bicarbonate was added to the cells, and they were lysed at 4°C under 35,000 psi for 45 minutes. 100 µg of protein was isolated for digestion using an acetone precipitation. After removing acetone, samples were reduced and alkylated, and sequence grade Lys-C/Trypsin (Promega) was used to enzymatically digest the extracted protein. All digestions were carried out in the Barocycler NEP2320 at 50°C under 20,000 psi for 1 hour. Digested samples were cleaned over C18 spin columns (Nest Group) and dried. Resulting pellets were resuspended in 97% purified water/3% acetonitrile (ACN)/0.1% formic acid (FA).

### LC-MS

The samples were analyzed using the Dionex UltiMate 3000 RSLC Nano System coupled to the Q Exactive™ HF Hybrid Quadrupole-Orbitrap MS (Thermo Scientific). Peptides were loaded onto a trap column (20 µm × 350 mm) and washed using a flow rate of 5 µl/minute with 98% purified water/2% ACN/0.01% FA. The trap column was then switched in-line with the analytical column after 5 minutes. Peptides were separated using a reverse phase Acclaim PepMap RSLC C18 (75 µm × 15 cm) analytical column using a 120 minute method at a flow rate of 300 nl/minute. Mobile phase A consisted of 0.01% FA in water and a mobile phase B consisted of 0.01 % FA in 80% ACN. The linear gradient started at 5% B and reached 30% B in 80 minutes, 45% B in 91 minutes, and 100% B in 93 minutes. The column was held at 100% B for the next 5 minutes before being brought back to 5% B and held for 20 minutes. Sample was injected into the QE HF through the Nanospray Flex™ Ion Source fitted with an emission tip from Thermo Scientific. Data acquisition was performed monitoring the top 20 precursors at 120,000 resolution with an injection time of 100 milliseconds.

### Proteomics data processing

Raw files obtained from the QExactive were uploaded using MaxQuant version 1.5.5.1 [97]. The following settings were applied in MaxQuant: Label free standard analysis using a multiplicity of 1. Label free quantitation (LFQ) was performed using a minimum ratio count of 2 with fast LFQ. Peptides were re-quantified and matched. Digestion was set to trypsin and Lys-C using a max missed cleavage of 2. The Orbitrap parameter was selected with a first search peptide tolerance of 20 ppm and a main search peptide tolerance of 4.5 ppm. Individual peptide mass tolerances were turned on with a centroid match tolerance of 8 pm and a centroid half width of 35 ppm. Calibration was intensity independent with a minimum peak length of 2, a max charge of 7, and a minimum score of 70 for recalibration. Oxidation of methionine and N-terminal acetylation were selected as variable modifications with a max of five modifications per peptide. Iodoethanol was selected as a fixed modification and contaminants were not excluded during the initial quantification. The minimum peptide length was set to 7 and the max peptide mass was set to 4600 Da. For unspecified searches, the minimum peptide length was set to 8 and the max peptide length was set to 25. Unmodified unique and razor peptides were used for quantification. At the peptide match level, false discovery rate was set to 0.01. Second peptides were included and match between runs was turned on with a match window time of 1 minute and an alignment window time of 20 minutes. Peptides were identified using an annotated human proteome .fasta from the Uniprot database.

### Proteomics data analysis

The identified protein groups generated by the MaxQuant program were uploaded to the Perseus program version 1.5.3.0 [98]. Site only, reverse, and contaminant peptides were removed from the dataset and missing values were imputed using a normal distribution. Invalid values were then excluded. Empty columns were removed. The volcano plot function was used to identify proteins that were significantly changed using a *t*-test with a false discovery rate of 0.05 and an S_0_ of 0.1. A 1.5 log2 fold cutoff change was applied for Ingenuity Pathway Analysis (IPA, Qiagen).

### Angiogenesis tube formation assay

EA.hy926 cells were cultured as described earlier. 3 × 10^4^ cells were seeded per well into a clear 96 well plate with 50 µL of preformed matrigel media and treated with 1, 10, or 50 µM C3 or 0.1% or 0.5% DMSO in 1× DMEM, 10% FBS (ThermoFisher), and 1× anti-anti. Calcein was added following the manufacturer’s protocols (Cellbiolab) and tube formation images were captured using an inverted fluorescence microscope (Olympus, IX71).

### c-MYC binding site luciferase reporter assay

15,000 PC-3 cells were seeded per well in a white reflective 96 well plate and transfected next day using 200 ng of a c-MYC binding site luciferase (pBV-Luc wt MBS 1–4) reporter plasmid and 4 ng of cytomegalovirus (CMV)-LacZ (B-Gal) plasmid for six hours using DMRIE-C transfection reagent according to manufacturer’s instructions. pBV-Luc wt MBS1-4 was a gift from Bert Vogelstein (Addgene plasmid # 16564). Transfection reagent was removed and replaced complete media for an additional 18 hours. Cells were then treated with 1, 10, or 50 µM of C3 for 24 hours. Media was aspirated, 1× passive lysis buffer was added, and cells were freeze-thawed after being stored at –80°C. Luciferase activity detection was measured using the Luciferase Assay System (Promega) and a Glomax Discover luminometer (Promega).

### Statistics

Statistical analysis was performed using GraphPad Prism 6. All assays were run in triplicate with values shown as the mean ± SEM unless otherwise indicated. Student’s *t*-test was used for pairwise comparisons and one-way ANOVA was used for group comparisons with significance set at *p* < 0.05. In the case of multiple comparisons, the Holm-Sidak test was used or a false discovery rate was applied.

## SUPPLEMENTARY MATERIALS FIGURES



## Abbreviations

LR: Laminin Receptor
PEDF: pigment epithelium derived factor
VEGF: vascular endothelial growth factor
THBS1: thrombospondin-1
(MMP)-2 and -9: matrix metalloproteinase
(VEGFR2): vascular endothelial growth factor receptor 2
(TGFB): transforming growth factor beta
(TIMP2): tissue inhibitor of metalloproteinase 2
(CASP3): caspase-3
(BAX): bcl-2-associated x protein
(P21 (RAC1) Activated Kinase 1): Rho guanine nucleotide exchange factor 7 (PIXB) and PAK1
DMSO: dimethyl sulfoxide
CCK-8: cell counting assay-8
ATCC: American Type Culture Collection
NCI: National Cancer Institute
