# Supplementary material for: A dock derived compound against laminin receptor (37 LR) exhibits anti-cancer properties in a prostate cancer cell line model
Source: Oncotarget. 2017 Dec 13;9(5):5958–78. doi: 10.18632/oncotarget.23236 (PMC5814187; doi:10.18632/oncotarget.23236)
Supplement: Supplementary file 1 [file oncotarget-09-5958-s001.pdf]

A dock derived compound against laminin receptor (37 LR) exhibits anti-cancer properties in a prostate cancer cell line model

SUPPLEMENTARY MATERIALS

MATERIALS AND METHODS

Endogenous 37 LR expression levels in screened cell lines

PC-3, Endo, TC2-Ras, SH-SY5Y, and LNCAP cells were grown in 100 mm plates in complete media and lifted at ~90% confluency using 20 mM EDTA in 1× DPBS. Cells were pelleted, media was aspirated, and pellets were frozen at –80°C until required for analysis. Total protein was isolated using 1X RIPA buffer (Thermo Scientific) containing 1X Halt Protease Inhibitor (Thermo Scientific), 5 mM EDTA, and Phosphatase Inhibitor Cocktails 2 and 3 (Sigma Aldrich). Cells were lysed passively on ice for thirty minutes, sheared using a tuberculin syringe (BD) and supernatant was separated from cellular debris after a 30 minute 14,000 rpm spin at 4°C. Protein concentration was determined using the BCA assay (Thermo Scientific). 50 µg of protein was loaded onto a Bolt 4–12% Bis-Tris Plus gel (Life Technologies) and electrophoresed for 30 minutes at 200V. Proteins were transferred onto nitrocellulose using the iBlot2 system (Life Technologies). The blot was blocked for 1 hour at room temperature using 5% BSA in 1× PBS with 0.1% Tween-20 (Acros). 37 LR was probed using a rabbit polyclonal (Bioss, bs-0900R, 1:250) and beta actin was probed using a mouse monoclonal (ThermoFisher, MA5-15739, 1:5000). Goat anti-rabbit IgG secondary (Licor, 925-68021, 1:15,000) and goat anti-mouse IgG secondary (Licor, 925-32210, 1:15,000) were used to detect primary antibody and signal was detected using the Licor Odyssey CLx. Image Studio Lite (Licor, ver. 5.2) was used to quantify pixel intensity.

Treatment of PC-3 cells with exogenous PEDF

Recombinant PEDF (BioProductsMD) was resuspended in PEDF Buffer (0.2 M NaCl, 20 mM sodium phosphate, and 1 mM DTT; pH 6.4). 1,500 cells per well were seeded in a clear 96-well plate in RPMI-1640 with 1% anti-anti and 10% FBS and allowed to grow overnight. A 0-hour recording was taken using CCK-8 and compared to a 48-hour reading after incubating PC-3 cells with recombinant PEDF (0.1 µM to 1.6 µM) in RPMI-1640 with 1% anti-anti and 1% FBS. PEDF Buffer was used as a vehicle control. Each 48 hour value was divided by its respective 0 hour value and normalized to PEDF Buffer to obtain % cell viability.

Generation and characterization of TC2-Ras cells

The TC2-Ras cell line was generated by our group and is useful in that they are very aggressive in their growth and form tumors that are challenging to treat, hence the benefit of testing new compounds with it. As described in Materials and Methods, TC2-Ras were generated by transduction of parental cells TRAMP-C2 with a lentivirus expressing a Ras gene at MOI = 1. When assayed for growth rate *in vivo,* TRAMP-C2 (10^6) are very slow growing as compared to TC2-Ras (5×10^5) when implanted subcutaneously in 6–8 week old C57/BL6 male mice (Supplementary Figure 4A). As described in Materials and Methods, TC2-Ras also were transduced with MOI = 1 of a lentivirus expressing mouse androgen receptor (mAR). mAR activity was assayed *in vivo* by bioluminescence imaging. An adenoviral vector containing an AR-responsive cassette expressing reporter gene luciferase was used. The adenovirus was administered intravenously (2×10^8 pfu/i.v.) 3 days following subcutaneous TC2-Ras cell implantation (10^4 or 10^5, without mAR (-mAR control) or with mAR (+mAR) in C57/BL6 male mice (Supplementary Figure 2B–2C). Mice were injected intraperitoneally with 150 mg/kg Luciferin (10–20min incubation) followed by a 5 min signal acquisition using an IVIS100 imager (Caliper/PerkinElmer, Downers Grove, IL) and analyzed with LivingImage 3.1 software (Caliper/PerkinElmer), with signals reported as photons per second per cubic centimeter per steridian (p/sec/cm^2/sr).

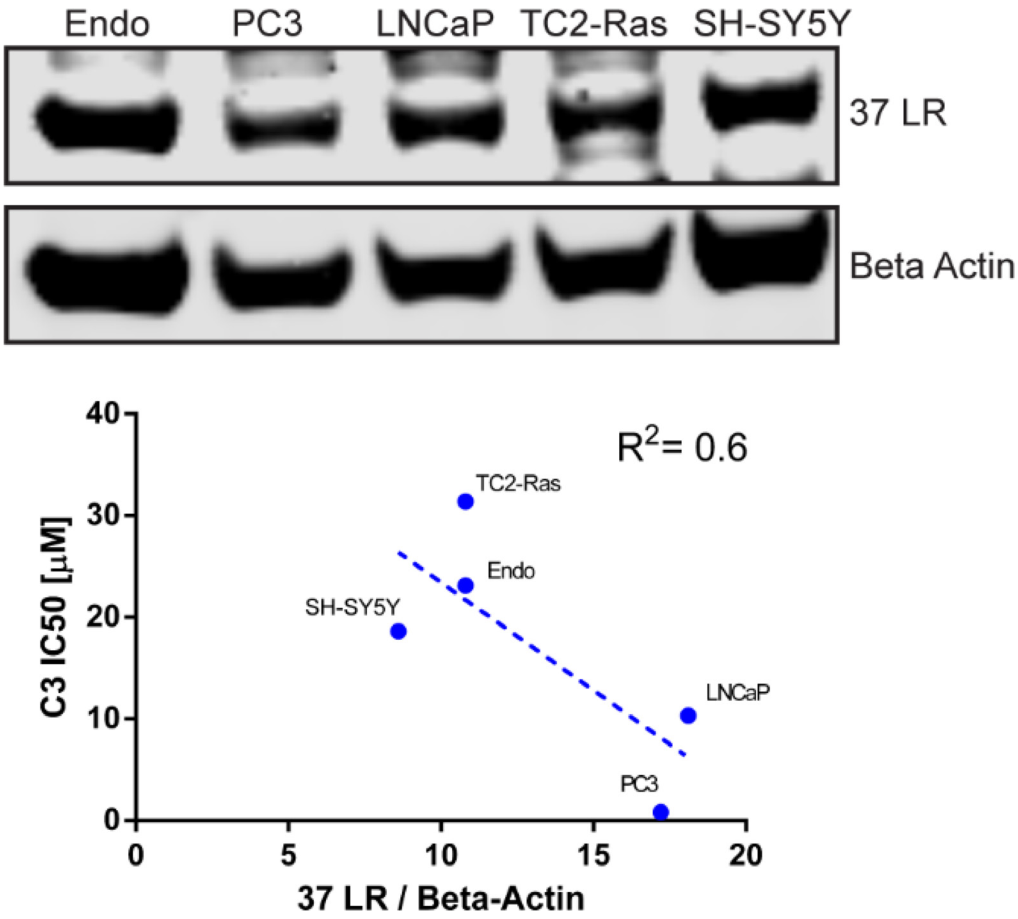

Supplementary Figure 1: Effectiveness of C3 correlates with endogenous 37 LR levels for the five cell lines screened for cell viability as assayed using a rabbit polyclonal against 37 LR. Beta-actin was used as a control. After normalizing 37 LR protein levels to beta-actin levels, the protein expression values were graphed against the C3 IC_{50} responses measured in Table 1. GraphPad Prism was used to generate a linear fit (*n* = 1).

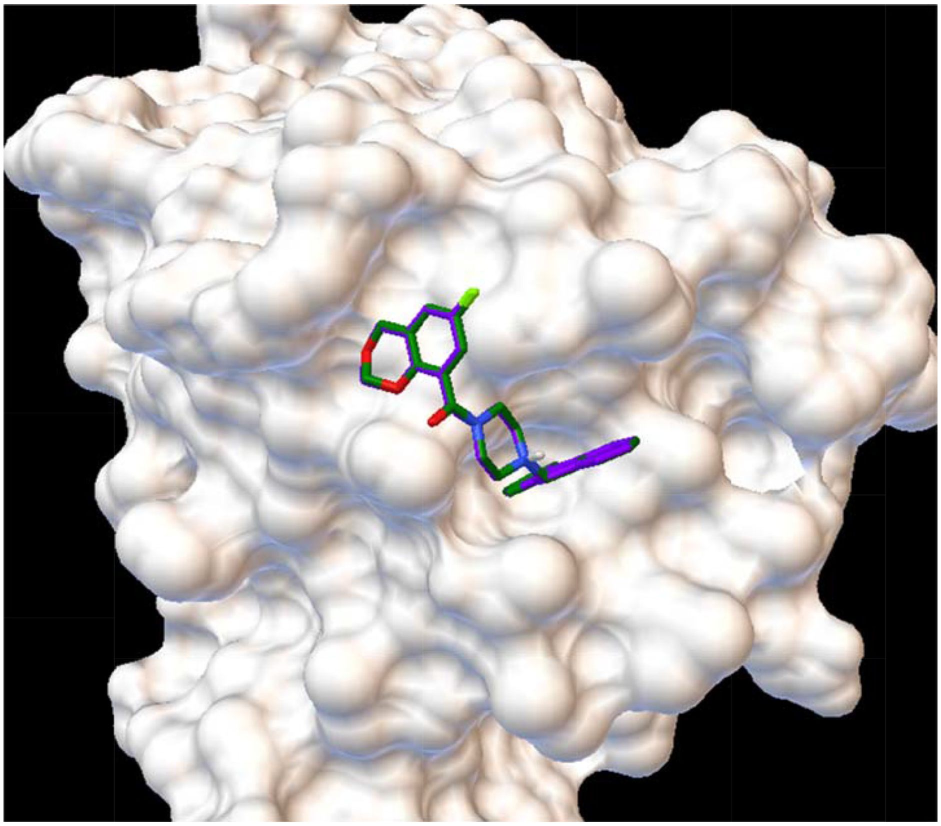

Supplementary Figure 2: His-169 protonation state does not alter C3 binding *in silico.* C3 was redocked to the LR crystal structure using an epsilon-protonated state His-169 or a delta-protonated state His-169 residue using Autodock Vina. A near complete superposition of the two poses is depicted in the image, demonstrating that His-169 protonation does not affect C3 binding to the LR pocket.

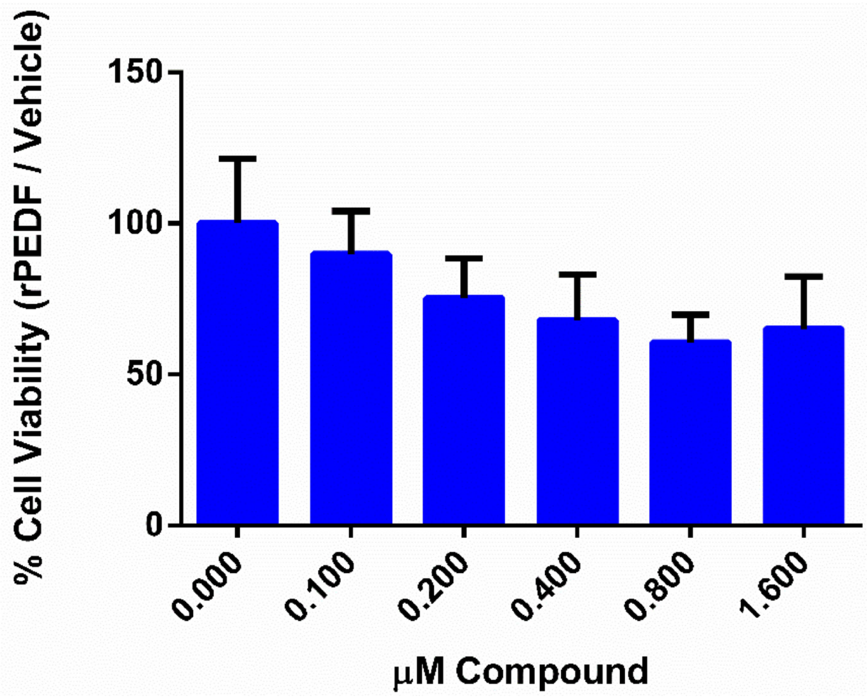

Supplementary Figure 3: PC-3 cell viability response to recombinant PEDF. PC-3 cells were treated with 0.1 to 0.6 µM rPEDF for 48 hours and cell viability was measured using the CCK-8 assay (n = 3, mean ± SEM). A one-way ANOVA was used for statistical analysis (n.s. $p = 0.11$) however the linear trend was significant (post test for linear trend, $p < 0.05$).

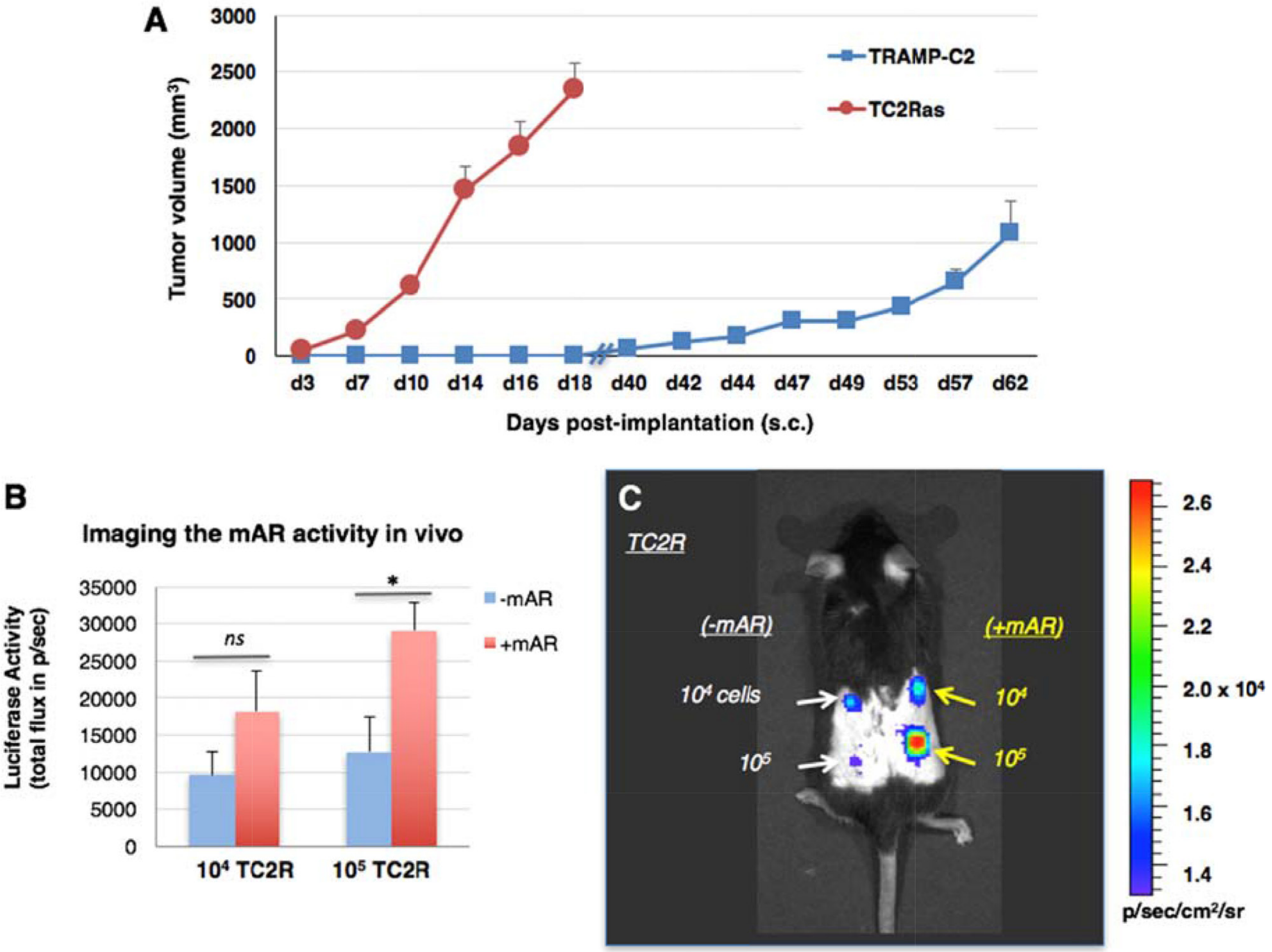

Supplementary Figure 4: TC2-Ras cell line characterization *in vivo*. The TC2-Ras cell line was generated by transduction of parental cells TRAMP-C2 with a lentivirus expressing a Ras gene at MOI = 1. (**A**) Comparison of subcutaneous growth rate *in vivo* between parental cell line TRAMP-C2 (10^6) and TC2-Ras (5×10^5) in 6-8 week old C57/BL6 male mice. (**B**) mAR activity was assayed *in vivo* by bioluminescence imaging. An adenoviral vector containing an AR-responsive cassette expressing reporter gene luciferase was administered intravenously (2×10^8 pfu/i.v.) 3 days following subcutaneous TC2-Ras cell implantation (10^4 or 10^5, without mAR (-mAR control) or with mAR (+mAR) in C57/BL6 male mice. Mice were injected intraperitoneally with 150 mg/kg Luciferin (10–20min incubation) followed by a 5 min signal acquisition using an IVIS100 imager (Caliper/PerkinElmer, Downers Grove, IL) and analyzed with LivingImage 3.1 software (Caliper/PerkinElmer). Statistics used a student's *t*-test to compare between mAR (−/+) groups for each cell concentration used. *ns*, $p > 0.05$ ($p = 0.21$); $^*p < 0.05$ ($p = 0.023$). (**C**) Representative image of a mouse that received either 10^4 or 10^5 TC2-Ras cells (–/+ mAR) and received reporter adenovirus intravenously as described in (B). Color bar, p/sec/cm^2/sr.
